# Educational and Equity Implications of Biomarker Testing and Targeted Therapy Access in NSCLC: Insights from the NIH All of Us Research Program

**DOI:** 10.1007/s13187-025-02775-z

**Published:** 2025-11-25

**Authors:** Patrick J. Kiel, Todd C. Skaar, David R. Foster, Karen Suchanek Hudmon, Michael A. Preston

**Affiliations:** 1 College of Pharmacy, Purdue University, West Lafayette, IN USA; 2 Indiana University School of Medicine, Indianapolis, IN USA; 3 Center for Health Excellence, Quality, and Innovation (CHEQI), Purdue University, Indianapolis, IN USA

**Keywords:** NIH All of US, Biomarker testing, Non-small cell lung cancer, Real-world evidence, Cancer education, Social determinants of health, All of Us

## Abstract

Precision oncology in non-small cell lung cancer (NSCLC) depends on biomarker testing and access to targeted therapies. However, disparities in testing and treatment highlight critical educational needs for patients, providers, and health systems. Using the NIH *All of Us* Research Program (2017–2022), we identified 287 patients with advanced NSCLC who received first-line carboplatin-based chemotherapy or targeted therapy. Social determinants of health (SDoH) including education, employment, retirement, and disability were evaluated as predictors of biomarker testing and targeted therapy receipt. Only 18% of patients had documented biomarker testing despite 45% minority representation in the *All of Us* cohort. Patients with any college education had significantly higher odds of receiving targeted therapy (OR 2.43, 95% CI 1.23–4.97), while retired patients were less likely to receive targeted therapy (OR 0.35, 95% CI 0.18–0.68). Disability was associated with increased biomarker testing (OR 2.82, 95% CI 1.19–6.51). Although TTD was longer for targeted therapies, incomplete biomarker documentation limited interpretation of outcomes. Findings underscore the need for enhanced cancer education strategies to improve biomarker literacy, guideline adherence, and equitable access to targeted therapy. Educational outreach should focus on retirees and patients with lower educational attainment, while provider training and system-level improvements are needed to strengthen precision oncology delivery.

**Supplementary Information:**

The online version contains supplementary material available at https://doi.org/10.1007/s13187-025-02775-z.

## Introduction

Non-small cell lung cancer (NSCLC) is the leading cause of cancer-related mortality, with the majority of patients presenting with advanced or metastatic disease, where five-year survival remains poor at 8–26% [1]. Precision oncology has transformed the management of NSCLC through the identification of actionable biomarkers (e.g., EGFR, ALK, ROS1, BRAF, METex14, RET, KRAS G12C, ERBB2, NTRK1/2/3), with targeted therapies consistently demonstrating improved outcomes compared to chemotherapy in pivotal clinical trials [2–4].

The National Comprehensive Cancer Network (NCCN) and the American Society of Clinical Oncology (ASCO) recommend broad molecular profiling for patients with advanced NSCLC to identify biomarkers and guide initial treatment decisions [2]. Hover, biomarker testing in real-world settings remains inconsistent, with rates ranging from 28 to 70% depending on practice setting, and many eligible patients never receive matched targeted therapies [5–9]. These gaps reflect both educational challenges from provider knowledge and guideline adherence to system-level inequities that limit patient access to precision oncology.

Real-world evidence (RWE) provides an opportunity to examine not only clinical outcomes but also the social determinants of health (SDoH) that shape cancer care [10–12]. The National Institutes of Health (NIH) *All of Us* Research Program offers a unique platform, integrating electronic health records, genomics, and survey data to study disparities across diverse populations [13, 14].

In this study, we leveraged *All of Us* data to examine biomarker testing and treatment patterns in advanced NSCLC, with a particular focus on education, employment, disability, and retirement as SDoH associated with testing and therapy receipt. Time-to-treatment discontinuation (TTD) was evaluated as a pragmatic endpoint to assess treatment durability [15], given incomplete mortality data in the dataset. By linking TTD with disparities in biomarker testing and therapy use, this work provides insights into the educational and equity implications of precision oncology delivery highlighting opportunities for provider training, patient outreach, and system-level improvements.

## Materials and Methods

### Data Source

This retrospective cohort study utilized data from the NIH *All of Us* Curated Data Repository, version 7, spanning January 2017 through June 2022 [13, 14]. The *All of Us* Research Program integrates electronic health records (EHRs), participant surveys, genomic data, physical measurements, and biospecimens from more than 400,000 participants nationwide, of whom ~ 45% identify as racial or ethnic minority. This diversity makes *All of Us* a unique platform for studying SDoH and cancer disparities in real-world settings. All of Us data are harmonized to the Observational Medical Outcomes Partnership (OMOP) Common Data Model, with diagnoses represented as condition_concept_ids and biomarker testing reflected in the procedure (CPT) and measurement (LOINC) tables. This harmonization ensures consistent ascertainment of testing across health systems.

### Study Population

We identified adults (> 18 years) with a diagnosis of advanced or metastatic NSCLC who received first-line systemic therapy. Patients were included if they received either: (a) a carboplatin-based regimen (pemetrexed or paclitaxel, with or without pembrolizumab), or (b) a targeted therapy consistent with NCCN guidelines. We focused on carboplatin (not cisplatin) because it was the predominant first-line doublet captured in *All of Us* during the period and reduces heterogeneity in dosing and toxicity patterns. Maintenance (e.g., pembrolizumab, pemetrexed) was not reliably separable from induction in EHR order data; TTD therefore reflects overall first-line treatment course rather than distinct induction/maintenance phases. Advanced NSCLC was defined as stage IV or metastatic disease in EHR problem lists or staging fields; histology was limited to adenocarcinoma or squamous cell carcinoma. Patients were excluded if they had more than one primary cancer, if treatment preceded the recorded diagnosis, if histology was not adenocarcinoma or squamous cell carcinoma, or if treatment duration was < 10 days.

### Biomarker Testing

Biomarker testing was identified using structured EHR data, with CPT4 codes capturing orders and billing for actionable genomic targets (e.g., EGFR, ALK, BRAF, KRAS, RET; Supplement Table S1). We included panel codes (81,445, 81,450, 81,455, 0037U), single-gene codes (81,210, 81,235, 81,275–81,276, 81,301), molecular pathology tier codes (81,400–81408), and unlisted codes (81,479, 81,599) to account for laboratory-developed tests. In addition, LOINC codes were used to capture laboratory test results for individual biomarkers (e.g., EGFR, KRAS, BRAF, ALK, NRAS, RET, MSI), complementing CPT-based ascertainment of test orders. Participants were classified as having “documented testing” if at least one relevant code was recorded. Because *All of Us* frequently captures incomplete or free-text results, we anticipated under-documentation, which itself represents an important educational and system-level barrier. Because NCCN/ASCO biomarker testing recommendations were largely consistent during 2017–2022, no adjustment for calendar year was applied [1, 16, 17].

### Social Determinants of Health (SDoH)

Self-reported survey data were used to categorize SDoH variables, including race/ethnicity, sex at birth, smoking history, disability status, education (any college vs. no college), employment (employed, unemployed, retired), marital status (married vs. not married), and income (< $75,000 vs. ≥ $75,000). Disability included self-reported functional limitations (e.g., vision, mobility, or self-care).

### Time-to-Treatment Discontinuation (TTD)

As a pragmatic outcome, we assessed TTD, defined as the interval between treatment initiation and the last recorded administration of systemic therapy. Patients without a documented discontinuation or death event were censored at their last recorded administration date or the database end date (July 1, 2022), whichever came first. Death information was derived from EHR problem lists and encounter discharge dispositions; however, incomplete capture of death dates in *All of Us* is a known limitation. TTD was selected because mortality data were incomplete, and it has been validated in real-world oncology studies as a proxy for treatment durability [15].

### Statistical Analysis

Descriptive statistics summarized baseline demographics, clinical characteristics, and SDoH variables. Group comparisons used chi-square or t-tests as appropriate. Logistic regression estimated odds ratios (ORs) for associations between SDoH variables and receipt of biomarker testing or targeted therapy. Kaplan–Meier and Cox proportional hazards models evaluated TTD by treatment type, with covariates including age, sex, race/ethnicity, smoking status, and biomarker testing. Missing values were retained as separate categories per *All of Us* policy to preserve privacy and sample size. Analyses were conducted in R version 4.4.0.

## Results

### Patient Characteristics

A total of 287 patients with advanced NSCLC receiving first-line therapy were identified (Table 1). The median age differed significantly between treatment groups, with patients receiving targeted therapy being younger (62.4 years) compared to those treated with carboplatin-based regimens (68.2 years; *p* < 0.001). Most participants were White and non-Hispanic. Smoking history was more common in the carboplatin group (74%) compared with the targeted therapy group (40%; *p* < 0.01). Documentation of biomarker testing was limited, observed in fewer than 20% of patients overall (Table 1).

**Table 1:** Baseline Characteristics

Characteristic	Carboplatin-Based Therapy (*N* = 162)	Targeted Therapy (*N* = 125)	*p*-value
**Age**, years (Median [Min, Max])	68.2 [36.9, 86.2]	62.4 [28.1, 89.4]	< 0.001
**Race**, n (%)			0.400
White	121 (74%)	94 (75%)	
Black or African American	41 (25%)	31 (24%)	
More than one population, Unknown, or Asian	—	—	
**Ethnicity**, n (%)			0.095
Hispanic or Latino, or Unknown	< 32	< 25	
Not Hispanic or Latino	> 130	> 100	
**Smoking history**, n (%)			0.002
Yes	120 (74%)	50 (40%)	
No	42 (25%)	75 (60%)	
**Biomarker Testing**, n (%)	Obscured, combined across groups: < 20%	—	0.060

* Wilcoxon Rank-Sum Test used for Age (not normally distributed); Pearson’s chi-Squared Test or Fisher’s Exact Test used for categorical variables; **Counts < 20 have been obscured to comply with NIH *All of Us* Data Policy. Supplementary Table S4 for regimen distributions

### Treatment Outcomes

TTD was significantly longer in patients treated with targeted therapies compared to those receiving carboplatin-based chemotherapy (median 11.5 vs. 3.3 months; hazard ratio 0.65, 95% CI 0.51–0.84; *p* < 0.001) (Supplement).

### Social Determinates of Health

Disparities in treatment patterns were associated with several SDoH variables (Table 2). A greater proportion of patients with disabilities were observed in the carboplatin group (22%) compared with the targeted therapy group (< 20%; *p* = 0.013). College education was more common among targeted therapy recipients (79%) compared with those on carboplatin-based regimens (66%; *p* = 0.020). Employment status also differed: 34% of targeted therapy patients were employed versus 18% of those on carboplatin, while retirement was more common in the carboplatin group (60% vs. 42%; *p* = 0.009). Marital status and income distributions did not significantly differ between groups (*p* > 0.10).

**Table 2:** Social Determinants of Health

Characteristic	Carboplatin-Based Therapy (*N* = 162)	Targeted Therapy (*N* = 125)	*p*-value
**Disability, n (%)**			0.013
No	126 (77%)	> 95	
Yes	36 (22%)	< 30	
**Education, n (%), Any college**			0.020
No	55 (34%)	26 (20%)	
Yes	107 (66%)	99 (79%)	
**Employment Status, n (%)**			0.009
Employed	30 (18%)	43 (34%)	
Retired	98 (60%)	53 (42%)	
Unemployed or Missing	34 (21%)	29 (23%)	
**Marital Status, n (%)**			0.108
Married	78 (48%)	73 (58%)	
Not married	84 (51%)	52 (41%)	
**Income, n (%)**			0.119
< $75,000	82 (50%)	58 (46%)	
≥ $75,000	45 (27%)	> 30	
Missing	35 (21%)	< 30	

### Regression Models

**Fig. 1:**
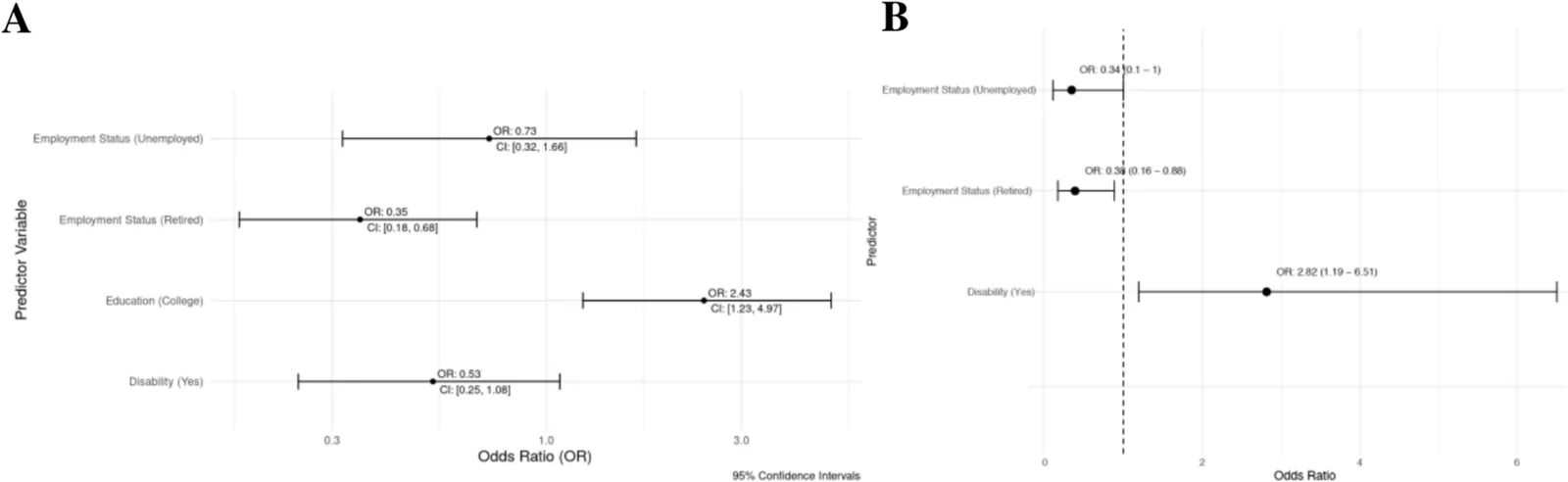
**A**. Targeted Therapy Receipt Associated with Social Determinants of Health. **B**. Documented Biomarker Testing Associated with Social Determinants of Health

Multivariable models confirmed these associations. Patients with any college education had more than twice the odds of receiving targeted therapy (OR 2.43, 95% CI 1.23–4.97) (Fig. 1a). Retired patients had significantly lower odds of targeted therapy (OR 0.35, 95% CI 0.18–0.68). In the biomarker testing model, disability was associated with higher odds of documented testing (OR 2.82, 95% CI 1.19–6.51), while retirement again predicted lower odds (OR 0.38, 95% CI 0.16–0.88) (Fig. 1b). Income and marital status were not significantly associated with either targeted therapy or biomarker testing.

## Discussion

This study leveraged the NIH *All of Us* Research Program to evaluate real-world treatment patterns, biomarker testing, and SDoH among patients with advanced NSCLC. As expected, targeted therapy was associated with significantly longer TTD, reflecting the fixed cycle structure of carboplatin-based chemotherapy compared with the continuous administration of oral targeted agents. However, the most striking finding was the under-documentation of biomarker testing (< 20%) and the disparities in testing and therapy access associated with education, disability, and retirement status.

Our results highlight important educational and equity implications in the care of patients with NSCLC. We observed that participants with a college education were more likely to receive targeted therapy, indicating that health literacy and patient advocacy may influence treatment access. In contrast, retired patients were less likely to undergo biomarker testing or receive targeted therapy. This may indicate a need for targeted outreach to older adults and those outside of the workforce. Disability was associated with higher odds of biomarker testing, which may reflect heightened recognition of functional limitations in care planning. Further study is necessary to confirm this relationship. These observations point to gaps in both provider adherence to guidelines and patient access to biomarker literacy resources. These findings along with Bouchard et al., underscore that addressing disparities in precision oncology may require community-driven educational interventions that go beyond the clinic, leveraging outreach and engagement infrastructures to tackle broader social determinants of health [18].

Financial barriers must be considered in relation to biomarker testing. Insurance coverage data were not available in the *All of Us* dataset, which limits our ability to assess payer-related barriers. However, in our cohort, income was not significantly different between those who did and did not receive biomarker testing but we cannot exclude that cost and coverage considerations may contribute to the low overall testing rates observed. Although these variables could not be directly analyzed, prior literature suggests that differences in reimbursement policies and out-of-pocket costs may influence access to precision oncology [19]. Future research linking *All of Us* to claims data would allow for a more complete assessment of the role of insurance and financial barriers in shaping equitable biomarker testing and treatment access.

The *All of Us* dataset’s diversity is a strength, but several limitations must be acknowledged. First, incomplete capture of biomarker testing likely underestimated true testing rates [20, 21]. While CPT/LOINC codes can be used to identify testing, many biomarker reports are stored as free text or external pdf documents. This limitation reflects documentation and informatics gaps that carry important educational implications for providers and health systems. Although SNOMED concepts can encode mutation findings (e.g., ‘EGFR mutation present’), these fields were inconsistently populated in All of Us and therefore were not used in this analysis. Our results should thus be interpreted as reflecting documented biomarker testing in structured EHR fields, rather than absolute testing rates. This framing emphasizes that our findings capture gaps in documentation fidelity as much as utilization, reinforcing the exploratory nature of the analysis.

Documentation of oral targeted therapies posed additional challenges. Dispense dates and days’ supply were not consistently available in *All of Us*. We therefore relied on last recorded administration or order dates to define treatment end. This pragmatic approach may under- or over-estimate true oral therapy duration, highlighting the need for better structured pharmacy data and provider training in documentation practices.

Our analyses were descriptive and exploratory, not causal. We did not apply propensity score matching or reweighting methods, as *All of Us* lacks consistent capture of critical prognostic variables such as ECOG performance status, cancer staging, or comprehensive comorbidity indices. These covariates balance treatment arms would risk introducing bias. As with the absence of ECOG, the most well-established prognostic factor in cancer, these gaps underscore the limitations of current real-world datasets for causal inference. Future work may leverage target-trial emulation and causal inference frameworks to more rigorously assess treatment effects once data completeness improves.

Although our cohort of 287 patients is reasonable for an exploratory analysis, the sample size limits precision for detecting small-to-moderate effects, and subgroup analyses should be interpreted with caution. Nonetheless, the consistent direction of key findings such as the influence of education and retirement on testing and therapy access do suggest meaningful disparities that warrant educational intervention. Additional consideration should be given to population representativeness. While the overall *All of Us* cohort includes ~ 45% racial and ethnic minority participants, our analytic subset was restricted to patients with advanced NSCLC who received systemic therapy, yielding a demographically different group. This enrichment step likely explains divergence from *All of Us* population benchmarks.

While TTD is a pragmatic endpoint validated in real-world oncology studies, it is not a substitute for overall survival. Death dates in *All of Us* come from problem lists and encounter dispositions, which are incompletely captured. Fewer than one-quarter of patients in our sample had a documented death despite a 54-month follow-up, underscoring a major limitation to survival analysis. Linking to external death indices will be essential to strengthen survival analyses [22].

These findings underscore the opportunity for cancer education to close persistent gaps in precision oncology. Strategies may include provider-focused education to improve ordering and structured documentation of biomarker testing into EHRs. Increased patient education campaigns to enhance awareness with self-advocacy, and community outreach tailored to populations most at risk of being underserved in order to improve patient health literacy. Improving biomarker literacy will require targeted, accessible interventions such as community-based outreach, patient navigator programs, and plain-language educational materials designed for individuals with limited health literacy. Housten et al. highlight interventions that incorporate simplified materials, teach-back methods, and multimedia approaches can effectively improve comprehension and engagement among cancer patients [23]. With the advent of artificial intelligence, innovative approaches such as AI-based chatbots have been proposed to deliver personalized, on-demand cancer education and enhance patient understanding of complex information and should be considered in these complex disease states [24]. Policies that integrate SDoH into oncology workflows to target retired individuals and patients with limited formal education are critical to advancing equitable delivery of precision oncology. Unmeasured factors such as health literacy, geographic access to oncology services, and provider practice variation may also have influenced the observed disparities and warrant further elucidation. These disparities in biomarker testing and targeted therapy access should be understood within the broader context of SDoH, which requires a holistic approach that integrates cultural, social, and systemic factors into cancer education and care delivery [25]. The *All of Us* scale and diversity uniquely position it to generate hypotheses about equity gaps and documentation gaps that directly inform provider training, health literacy initiatives, and system-level cancer education strategies.

## Conclusion

In summary, while targeted therapies extend treatment duration in advanced NSCLC, disparities in biomarker testing and targeted therapy access remain, shaped by education, retirement, and disability status. These findings highlight urgent opportunities for cancer education at the provider, patient, and system levels to improve biomarker literacy, guideline adherence, and equitable access to precision oncology.

## Supplementary Information

Below is the link to the electronic supplementary material.

Supplementary file1 (DOCX 2595 KB)

## Declarations

### Statement of Educational Relevance (required by JCE)

This study highlights how social determinants of health influence biomarker testing and targeted therapy access in NSCLC. Findings identify educational gaps for patients, providers, and health systems, underscoring opportunities for targeted cancer education initiatives to promote equitable precision oncology care.
