# Supplementary material for: Educational and Equity Implications of Biomarker Testing and Targeted Therapy Access in NSCLC: Insights from the NIH All of Us Research Program
Source: J Cancer Educ. 2025 Nov 25;41(5):979–85. doi: 10.1007/s13187-025-02775-z (PMC13755008; doi:10.1007/s13187-025-02775-z)
Supplement: Supplementary file 1 [file 13187_2025_2775_MOESM1_ESM.docx]

**Supplementary Appendix**

**Supplementary appendix to: Kiel PJ. Educational and Equity Implications of Biomarker Testing and Targeted Therapy Access in NSCLC: Insights from the NIH *All of Us* Research Program**

**Table of Contents**

**Figure S1 Flowchart for Inclusions and Exclusions in the Cohort Building…………………2**

**Table S1. Biomarker Screener……...………………………………………………………………...3**

**Table S2. Targeted Therapy Arm According to NCCN Guidelines Between 2018-2025.…...4**

**Table S3. Carboplatin-based Chemotherapy Arm Options…...…………………………………4**

**Table S4. Distribution of Carboplatin-Based Therapy.………………….………………………..5**

**Table S5. Distribution of Targeted Therapy…………………...…………………………………...5**

**Figure S2. Time-to-Treatment Discontinuation in First-line Advanced NSCLC……….….6**

**Figure S3. TTD, Carboplatin Double, Triplet, and Targeted Therapy………………………….7**

**Figure S1.** Flowchart for Inclusions and Exclusions in the Cohort Building.

**Table S1. CPT Code Biomarker Screener**

| Test Name | CPT Code |
| --- | --- |
| BRAF (B-Raf proto-oncogene, serine/threonine kinase) (eg, colon cancer, melanoma), gene analysis, V600 variant(s) | 81210 |
| EGFR (epidermal growth factor receptor) (eg, non-small cell lung cancer) gene analysis, common variants (eg, exon 19 LREA deletion, L858R, T790M, G719A, G719S, L861Q) | 81235 |
| KRAS (Kirsten rat sarcoma viral oncogene homolog) (eg, carcinoma) gene analysis; additional variant(s) (eg, codon 61, codon 146) | 81276 |
| KRAS (Kirsten rat sarcoma viral oncogene homolog) (eg, carcinoma) gene analysis; variants in exon 2 (eg, codons 12 and 13) | 81275 |
| Microsatellite instability analysis (eg, hereditary non-polyposis colorectal cancer, Lynch syndrome) of markers for mismatch repair deficiency (eg, BAT25, BAT26), includes comparison of neoplastic and normal tissue, if performed | 81301 |
| Molecular pathology procedure, Level 1 | 81400 |
| Molecular pathology procedure, Level 2 | 81401 |
| Molecular pathology procedure, Level 3 | 81402 |
| Molecular pathology procedure, Level 4 | 81403 |
| Molecular pathology procedure, Level 5 | 81404 |
| Molecular pathology procedure, Level 6 | 81405 |
| Molecular pathology procedure, Level 7 | 81406 |
| Molecular pathology procedure, Level 8 | 81407 |
| Molecular pathology procedure, Level 9 | 81408 |
| Targeted genomic sequence analysis panel, hematolymphoid neoplasm or disorder, DNA analysis, and RNA analysis when performed, 5-50 genes (eg, BRAF, CEBPA, DNMT3A, EZH2, FLT3, IDH1, IDH2, JAK2, KRAS, KIT, MLL, NRAS, NPM1, NOTCH1), interrogation for sequenc | 81450 |
| Targeted genomic sequence analysis panel, solid organ neoplasm, DNA analysis, and RNA analysis when performed, 5-50 genes (eg, ALK, BRAF, CDKN2A, EGFR, ERBB2, KIT, KRAS, NRAS, MET, PDGFRA, PDGFRB, PGR, PIK3CA, PTEN, RET), interrogation for sequence varian | 81445 |
| Targeted genomic sequence analysis panel, solid organ or hematolymphoid neoplasm, DNA analysis, and RNA analysis when performed, 51 or greater genes (eg, ALK, BRAF, CDKN2A, CEBPA, DNMT3A, EGFR, ERBB2, EZH2, FLT3, IDH1, IDH2, JAK2, KIT, KRAS, MLL, NPM1, NR | 81455 |
| Targeted genomic sequence analysis, solid organ neoplasm, DNA analysis of 324 genes, interrogation for sequence variants, gene copy number amplifications, gene rearrangements, microsatellite instability and tumor mutational burden | 0037U |
| Unlisted molecular pathology procedure | 81479 |
| Unlisted multianalyte assay with algorithmic analysis | 81599 |

Note: CPT4 codes capture biomarker test orders and billing. Unlisted codes (81479, 81599) were retained to capture laboratory-developed tests. LOINC codes were used in the primary analysis to complement CPT by capturing actual laboratory results for specific biomarkers (e.g., EGFR, KRAS, BRAF, ALK, NRAS, RET, MSI).

**Table S2. Targeted Therapy Arm (According to NCCN Guidelines Between 2018-2025)**

| **Mutation** | **Targeted Therapy 1L Option** |
| --- | --- |
| **EGFR Exon 19 Deletion or Exon 21 L858R** | Afatinib, Erlotinib, Dacomitinib, Gefitinib, Osimertinib, Erlotinib + ramucirumab, Erlotinib + bevacizumab, Amivantamab-vmjw + lazertinib |
| **EGFR S768I, L861Q, and/or G719X** | Afatinib, Erlotinib, Dacomitinib, Gefitinib, Osimertinib |
| **ALK Rearrangement** | Alectinib, Brigatinib, Ceritinib, Crizotinib, Ensartinib, Lorlatinib |
| **ROS1 Rearrangement** | Crizotinib, Entrectinib, Repotrectinib |
| **BRAF V600E Mutation** | Dabrafenib/trametinib, Encorafenib/binimetinib, Dabrafenib, Vemurafenib |
| **NTRK1/2/3 Gene Fusion** | Larotrectinib, Entrectinib, Repotrectinib |
| **MET Exon 14 Skipping Mutation** | Capmatinib, Crizotinib, Tepotinib |
| **RET Rearrangement** | Cabozantinib, Selpercatinib, Pralsetinib |
| **ERBB2 (HER2) Mutation** | Fam-trastuzumab deruxtecan-nxki, Ado-trastuzumab emtansine |

**Table S3. Carboplatin-based Chemotherapy Arm**

**Carboplatin-based Chemotherapy Arm**

-Carboplatin + Pemetrexed

-Carboplatin + Pemetrexed + Pembrolizumab

-Carboplatin + Paclitaxel

-Carboplatin + Paclitaxel + Pembrolizumab

Monitoring During Initial Therapy: Response assessment after 2 cycles, then every 2–4 cycles with CT of known or high-risk sites of disease (eg, chest, abdomen, pelvis) with or without contrast or when clinically indicated. Timing of CT scans within Guidelines parameters is a clinical decision.

Monitoring During Subsequent Therapy or Maintenance Therapy: Response assessment with CT of known or high-risk sites of disease (eg, chest, abdomen, pelvis) with or without contrast every 6–12 weeks. Timing of CT scans within Guidelines parameters is a clinical decision.

**Table S4. Distribution of Carboplatin-Based Therapy**

| **Platinum-Based Regimens** | **Count** | **Percent** | **Mean Months Duration** | **SD** | **Median Months Duration** | **IQR** |
| --- | --- | --- | --- | --- | --- | --- |
| Carboplatin/Paclitaxel | 101 | 62.40% | 10.2 | 21.9 | 2.7 | 1.2-5.1 |
| Carboplatin/Pemetrexed | 36 | 22.20% | 16.7 | 19.5 | 3.9 | 2.1-28 |
| Carboplatin + (Pemetrexed or Paclitaxel) + Pembrolizumab | 25 | N/A | N/A | N/A | N/A | N/A |

Doublet, Carboplatin + (Pemetrexed or Paclitaxel); Triplet, Carboplatin +(Pemetrexed or Paclitaxel) + Pembrolizumab

**Table S5. Distribution of Targeted Therapy**

| **Targeted Therapy** | **Cases** | **Percent** | **Mean Months**  **Duration** | **SD** | **Median Months Duration** | | | **IQR** |
| --- | --- | --- | --- | --- | --- | --- | --- | --- |
| cabozantinib | 31 | 24.8 | 15.3 | 19.7 | 7.8 | | | 3-26 |
| Other Drugs (Collapsed) | 94 | 75.2 | 17.6 | 21.7 | 9.9 | | | 3.9-23.2 |
| osimertinib | * |  |  |  |  | | |  |
| erlotinib | * |  |  |  |  | | |  |
| dabrafenib/trametinib | * |  |  |  |  | | |  |
| crizotinib | * |  |  |  |  | | |  |
| alectinib | * |  |  | | |  |  |  |
| afatinib | * |  |  | | |  |  |  |
| ado-trastuzumab emtansine | * |  |  | | |  |  |  |
| binimetinib | * |  |  | | |  |  |  |
| ceritinib | * |  |  | | |  |  |  |
| larotrectinib | * |  |  | | |  |  |  |
| lorlatinib | * |  |  | | |  |  |  |
| selpercatinib | * |  |  | | |  |  |  |
| sotorasib | * |  |  | | |  |  |  |
| vemurafenib | * |  |  | | |  |  |  |

***Fewer than 20 participants obscured due to NIH AoU Policy**

**Figure 1.** **Time-to-Treatment Discontinuation in First-line Advanced NSCLC**

**Figure S3. Time to Treatment Discontinuation, Carboplatin-Doublet, Carboplatin-Triplet, and Targeted Therapy.**
